# Exacerbation of COVID-19 mortality by the fragmented United States healthcare system: A retrospective observational study

**DOI:** 10.1016/j.lana.2022.100264

**Published:** 2022-05-12

**Authors:** Travis Campbell, Alison P. Galvani, Gerald Friedman, Meagan C. Fitzpatrick

**Affiliations:** a Department of Economics, Southern Oregon University, 1250 Siskiyou Blvd., Ashland, OR 97520; b Center for Infectious Disease Modeling and Analysis (CIDMA), Yale School of Public Health, New Haven, CT, USA; c Department of Economics at the University of Massachusetts at Amherst, Amherst, MA, USA; d Center for Vaccine Development and Global Health, University of Maryland School of Medicine, Baltimore, MD, USA

## Abstract

**Background:**

Before widespread vaccination, the United States was disproportionately affected by COVID-19 with a mortality rate several times that of other affluent societies. Comparing regions with different rates of health insurance, we assess how much of this excess mortality may be due to the relatively large population without health insurance.

**Methods:**

We use daily surveillance data from the US Centers for Disease Control and Prevention (CDC) stratified by region, age group, gender, and race in regression analysis of daily COVID-19 cases, hospitalization, and mortality. COVID-19 data have been matched with structural characteristics of the region including average proportion with health insurance. As checks, we have estimated regressions for different time periods, different groups of states, and by comparing adjacent counties between states with and without Medicaid expansion.

**Findings:**

Groups with lower health insurance coverage had significantly higher mortality as well as greater case counts and hospitalization. Early in the pandemic, they were also less likely to be tested for COVID-19. Applying our regression estimates, we estimate that had there been full health insurance coverage of the population, there would have been 60,000 fewer deaths, 26% of the total death toll in the period of this analysis.

**Interpretation:**

Our study demonstrates that a significant share of COVID-19 mortality in the United States, and much of the excess mortality in the United States compared with other countries, is due to our reliance on a system of market-driven healthcare. Providing universal insurance coverage should be part of our campaign to reduce COVID-19 mortality. It also suggests that these concerns should not be restricted to COVID-19 but apply across all diseases, contributing to many unnecessary deaths in the United States each year even apart from the COVID-19 pandemic.

**Funding:**

This study was supported by grants from the NSF (Expeditions grant 1918784), and the NIH (1R01AI151176-01 and 5K01AI141576).

**Research in context**

**Evidence before this study**

Health insurance has been shown to be effective in reducing mortality rates in the United States. It has been hypothesized that incomplete health insurance coverage might also have exacerbated the spread and severity of COVID-19. However, the impact of insurance coverage on COVID-19 cases, hospitalization, and mortality has not been quantitatively evaluated.

**Added value of this study**

This is the first study to quantify the impact of health insurance on the population-level disease and mortality burden of COVID-19. Using two independent approaches, we found that low insurance coverage is associated with more COVID-19 cases, hospitalizations, and deaths. If there had been universal health coverage, we estimate that 26% of COVID-19 mortality could have been averted through February 2021.

**Implications of all the available evidence**

In a pandemic such as COVID-19, universal health care can save lives as well as prevent substantial morbidity. Combined with the evidence of its life-saving impact prior to the emergence of COVID-19, there is an urgent need for the United States to move towards a system that ensures healthcare for all.

Alt-text: Unlabelled box

## Background

The United States has been disproportionately affected by COVID-19. With only 4% of the world's population, the cumulative mortality attributable to COVID-19 in the US through early 2021 is 15% of that of the global total. This disproportionate toll is magnified when comparing the US to similarly affluent countries and even more so if the comparison is made before the rollout of widespread vaccination. If the US had the per capita COVID-19 mortality through February 2021 of Canada or Germany, there would have been 200,000 fewer deaths. If the US had Australia or Japan's death rate, 400,000 lives would have been saved.^1^, ^2^, ^3^

Alone among advanced economies, many Americans are without health insurance. During a health crisis, the uninsured can be faced with the choice between prohibitively expensive medical bills and health risks from forgoing care. This dilemma is heightened by medical costs in the US that are unsurpassed globally. Even prior to the pandemic, nearly 20% of Americans had no regular source of health care, Americans had fewer physician visits than residents of other affluent countries, and they had shorter life expectancy.^4^^,^^5^ Life expectancy in the US also has disparities that reflect the extreme wealth inequality and relatively under-funded welfare state.^6^ In the US, the pandemic struck a population where many had constrained access to the diagnosis and treatment crucial to reducing transmission and case fatality rates.

Here we evaluate the repercussions of the US healthcare system on COVID-19 mortality. We demonstrate that a substantial component of the disparity between mortality from COVID-19 in the US and other affluent countries is due to the significant share of the US population without health insurance.

In our analysis of over 26 million cases, we estimated the impact of insurance coverage on COVID-19 mortality using daily individual-level data on COVID-19 cases, hospitalization, and mortality, as well as the delay between symptom onset and COVID-19 test results. Data were aggregated by state, gender, race, and age, and matched with demographic controls and health insurance coverage rates. We find that nearly 60,000 excess deaths from COVID-19, over 220,000 additional hospitalizations, and 2.9 million additional cases are associated with lack of health insurance. We also find that, prior to emergency appropriations that were enacted to cover the costs of testing, insurance coverage is associated with prompter testing following symptom onset during the initial phase of the pandemic.

Further, we explored the effect of Medicaid expansion on COVID-19 outcomes, comparing counties across state lines. We find that the impact of being in a state with Medicaid expansion compared to an adjacent country in a state without Medicaid expansion is consistent with our national-level findings regarding the effect of health insurance coverage.

## Methods

We obtained surveillance data from the US Centers for Disease Control and Prevention (CDC) delineating laboratory-confirmed cases of COVID-19 and their associated symptoms, comorbidities, date reported to CDC, date of symptom onset, date of positive test, hospitalization status, and mortality from January 1, 2020 through February 13, 2021 (Table 1). The dataset contains over 26 million cases and more than 225,000 deaths through February 13, 2021, corresponding to 73% of cases and 54% of deaths reported by CDC over the same timeframe.^7^ We stratified these data by the earliest of the three dates (symptom onset, date of positive test, or date reported to CDC), age, gender, race, and reporting state, and matched each stratum to its average insurance coverage over 2015–2019 using the Behavioral Risk Factors Surveillance Systems (BRFSS) survey. National coverage measured by BRFSS was 91.7%, similar to but slightly higher than the 90.8% measured by the 2019 American Community Survey.^8^ We extracted other characteristics of the stratum to use as controls, including additional demographics (marital status, education, and employment), self-reported health (smoking, drinking, physical health, and health in general), and the prevalence of comorbidities associated with heightened COVID-19 severity (obesity, hypertension, diabetes, respiratory disease, renal disorders, and cancer).^9^ We specifically chose BRFSS over other survey instruments due to this inclusion of comorbidity prevalence. We then applied a fixed-effects model to evaluate the association between insurance status and four key outcomes for each day within every state, gender, race, and age stratum: the number of reported cases, the number hospitalized, the number of deaths, the total number of days spent between symptom onset and test administration, and the average delay between symptom onset and test.

**Table 1:** Summary statistics for key variables in the CDC surveillance dataset.

Insurance coverage [unweighted, equal weight for all cells as in regressions]	0.890
Total Person-Days Between Symptom Onset and Test Administration	45,591,172
Total COVID-19 cases through February 13, 2020	25,942,037
Total Hospitalizations	1,207,878
Total COVID-19 deaths	225,210
Days	410
Strata	2,395

Accordingly, the regression specification follows:

(1) Y_c,a,s,r,t_ = α + βInsurance_c,a,s,t_ + X’_c,a,s,t_γ + δ_c_ + ζ_a_ + η_s_ + κ_r_ + θ_y_ + ἐ_c,a,s,t_

where Y is the COVID-19 outcome on day *t* for individuals in state *c* of age *a*, sex *s* and race *r*. α is a constant, β indicates a coefficient, Insurance denotes the 2015-2019 insurance rate for the state-race-age-sex stratum, δ_c_ denotes state fixed effects, ζ_a_ denotes age fixed effects, η_s_ denotes sex fixed effects, κ_r_ denotes race fixed effects, θ_y_ denotes daily time fixed effects, ἐ_,c,a,s,t_ is the error term, γ is a vector of coefficients for the control variables; X denotes a vector of controls. To evaluate the impact of particular controls on our results, we iteratively expanded the vector of controls to include the additional demographics, then self-reported health, then the prevalence of comorbidities. All regressions were unweighted. Standard errors were clustered by state-age-sex-race, as this is the level at which we observe insurance coverage.

We convert the estimated coefficients from the regressions to proportion changes:

(2) βˆ ∗ (G) ∗ (D) ∗ (1 − ¯I)/ ∑Y,

where βˆ is the estimated regression coefficient for the daily impact of stratum insurance rate on the clinical outcome Y, G is the total number of state-age-race-sex strata, D is the total number of days over the pandemic, ¯I_2019_ denotes the 2015-2019 average insurance coverage for the stratum, and ∑Y is the total events of each clinical outcome over every combination of strata.

To estimate the COVID-19 outcomes that were due to lack of insurance at a national level, we calculated the proportionate change in each outcome that arises when moving from 100% insurance coverage across all cells to 89%, with 100% representing universal coverage and 89% the unweighted average for insurance coverage across all included state-age-race-sex strata. We then used this proportion change as a multiplier on observed national COVID-19 outcomes to determine the number of COVID-19 cases, hospitalizations and deaths that are attributable to incomplete coverage.

### Sensitivity analyses

We explored the sensitivity of our analysis to alternative specifications in two areas: the timing of reports to the CDC, and geographic disparities in reporting. Due to potential delays in case reporting to CDC, data for later dates may be less complete than those for earlier periods. Additionally, the strength of association between insurance coverage and outcomes may have changed over time as the US pandemic response evolved. Therefore, we conducted the analysis as described above but using alternative cutoff dates for a case inclusion, spanning May 2020 through the end of February 2021.

Similarly, fewer cases and deaths are reported in the case surveillance data than are reported by the CDC. The difference between these two sources varies greatly by state. We therefore assess the robustness of the estimates to state-specific reporting vagaries by systematically omitting states according to increasing thresholds for completeness of reporting deaths.

### Contiguous counties analysis

**Figure 1:**
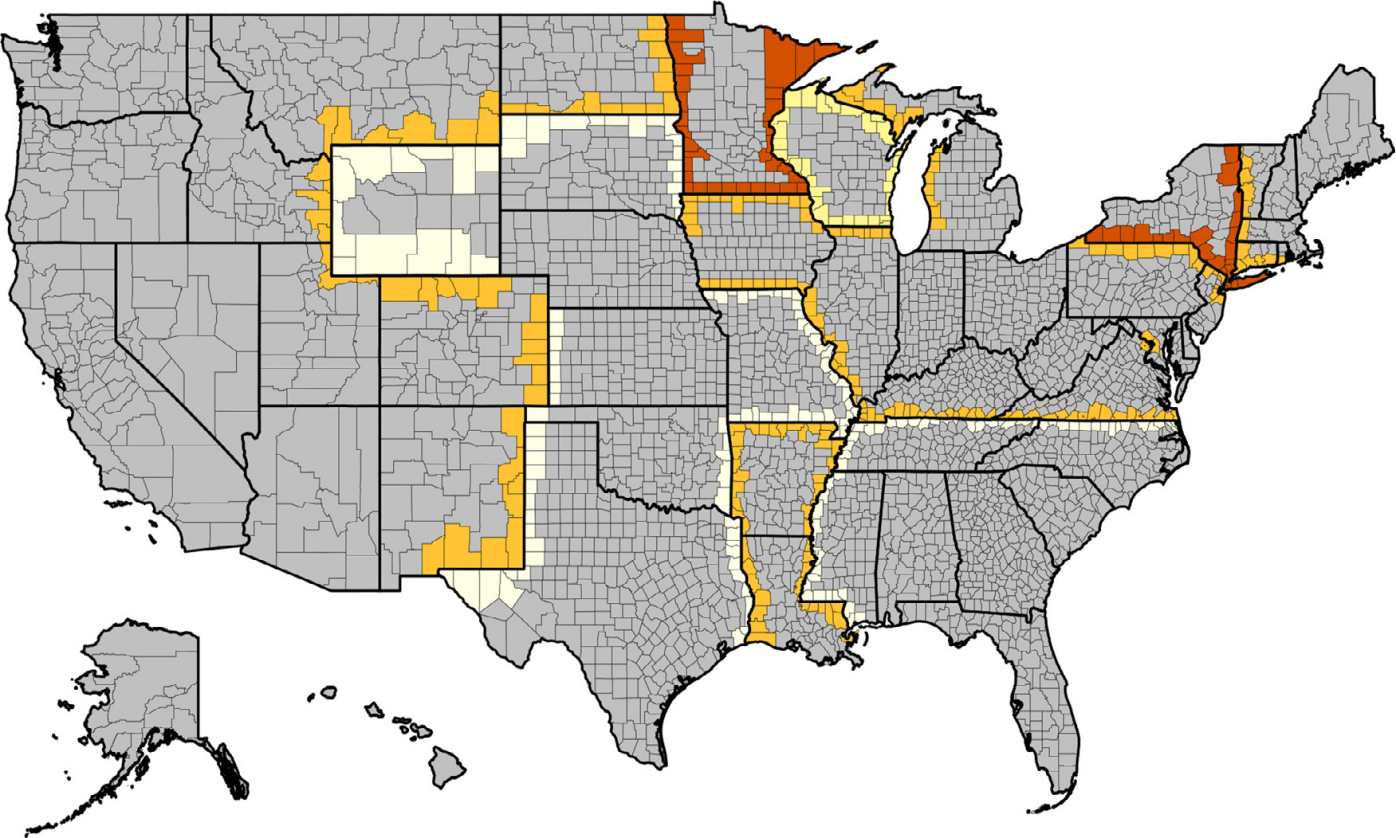
Map of counties included in the Medicaid expansion analysis. County color indicates the cutoff for Medicaid eligibility, as a multiplier on the federal poverty level (FPL): non-expansion (white), 100% (yellow), 138% (orange), 200% (red), 215% (dark red, District of Columbia only). Counties shaded gray do not border a state with a different Medicaid expansion policy, and were omitted from the analysis.

We also explored the robustness of our findings using an alternative approach that examined the impact of Medicaid expansion in particular, comparing COVID-19 outcomes across contiguous county pairs that straddle a border between states with different poverty thresholds for Medicaid eligibility. Medicaid expansion eliminates categorical restrictions on Medicaid and raises the income eligibility limit to 138% of the Federal Poverty Line (FPL). Expansion can produce abrupt changes in insurance coverage between otherwise similar counties, creating a natural experiment to estimate the impact of insurance coverage on COVID-19 outcomes.^10^ There are 8 states that have not approved Medicaid expansion, 3 states with poverty thresholds other than 138%, and 466 counties along the boundaries of these states, generating 942 contiguous county pairs (see Figure 1).

Our approach involves two stages. The first stage is a regression of the insurance rate within contiguous counties on an indicator for relatively generous Medicaid eligibility, and the second stage is a regression of COVID-19 health outcomes on the county-specific insurance rate as determined by the Small Area Health Insurance Estimates.^11^ We controlled for population, population density, household size, the percentage of population living in poverty, the percentage of population under 65, and urban/rural classification, political and COVID-19 variables including the number of COVID-19 tests administered, COVID-19 test positivity, the CDC Social Vulnerability Index, the COVID-19 Community Vulnerability Index, and the percent of population vaccinated over age 65. These variables were extracted from the CDC COVID-19 County Integrated View.^7^ We also controlled for the Republican vote share in the 2016 Presidential election.^12^

All analyses were conducted using STATA 16.

### Role of the funding source

The funders had no role in study design, data collection, data analysis, interpretation, writing of the report, or the decision to submit.

## Results

**Figure 2:**
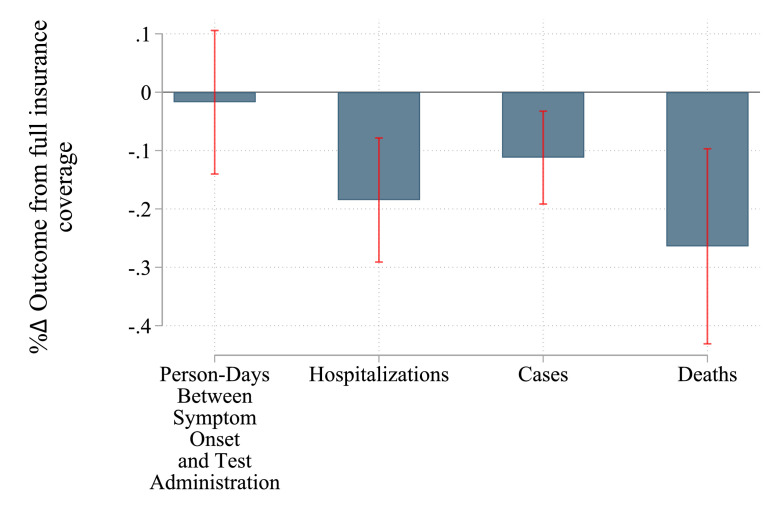
Effect on COVID-19 outcomes of moving from current health insurance rate to full coverage using the fixed-effects analysis. All states, through February 2021. The blue bars show the predicted effect as a percentage change in each outcome; the red lines show the 95% confidence intervals.

Among age, gender, race, and state strata, those with higher rates of health insurance coverage have substantially fewer deaths, hospitalizations, and cases. The association between health insurance coverage and testing delay is not statistically significant when using the full sample (see Table 2 and Figure 2).

**Table 2:** Impact of insurance coverage on four COVID-19 outcome variables based on panel data regressions in the CDC surveillance data set through February 13, 2021. The regression is given in Eq. (1); the proportion change on the insurance variable is from Eq. (2). The change in the outcome variables is calculated as the product of the proportion change and the number of recorded outcomes. Because the sample is not the entire population, the estimate for the effect of insurance on the entire population comes from applying the proportion change to the entire population at risk.

	Proportion Change	Total through Feb 13	Effect if all insured	Range (+/- 1 s.e.)
Person-days between symptom onset and test	-0.017 (s.e. 0.063)	45,591,172	(775,049)	(-3,692,884 - 2,142,785)
Cases	-0.112^⁎⁎⁎^ (s.e. 0.041)	25,942,037	(2,905,508)	(1,841,885 - 3,969,132)
Hospitalization	-0.185^⁎⁎⁎^ (s.e. 0.054)	1,207,878	(223,457)	(158,232 - 288,683)
Deaths	-0.264^⁎⁎⁎^ (s.e. 0.085)	225,210	(59,455)	(40,313 - 78,598)

Note: *p<0.10, **p<0.05, ***p<.01.

We compared the outcomes observed under the average insurance coverage of 89% for our strata with the counterfactual scenario of universal coverage. Lowering the insurance coverage from 100% in a stratum to 89% exacerbates mortality by over 26% (see Table 2 and Figure 2). At the national level, compared to a counterfactual of universal health insurance coverage for the US, this translates to 59,455 more COVID-19 deaths before February 13, 2021. This is nearly double the annual mortality due to lack of health insurance estimated for the pre-pandemic era.^13^

Insurance coverage was also strongly associated with higher COVID-19 caseloads and hospitalization. Reducing insurance coverage from 100% to 89% is associated with an 11% increase in cases and an 18% increase in hospitalizations. Consequently, the national insurance gap likely contributed to an excess of 3 million cases and 223,000 hospitalizations.

The sign and significance of our results are not sensitive to controlling for the strata's average demographic characteristics, self-reported health, or comorbidity prevalence (Table 3). Inclusion of the controls for comorbidity notably increases the magnitude of the estimates.

**Table 3:** Sensitivity of results to the inclusion of control variables. The proportion change in each outcome is presented based on an iteratively expanded inclusion of control variables. Fixed effects refers to the inclusion of state fixed effects, time fixed effects, age fixed effects, sex fixed effects, and race fixed effects. Demographic controls include demographic data beyond those accounted for by the fixed effects, in this case including marital status, college education (at least some), employment. Health controls include smoking, drinking, self-reported poor physical health days over past 30, and self-reported poor health in general. Comorbidity controls include obesity, hypertension, diabetes, respiratory system disease, renal disorders, and cancer (not skin cancer).

	Specification 1	Specification 2	Specification 3	Full Specification
Person-days between symptom onset and test	−0.046 (s.e. 0.056)	−0.023 (s.e. 0.054)	−0.04 (s.e. 0.055)	−0.017 (s.e. 0.063)
Cases	−0.106^⁎⁎⁎^ (s.e. 0.031)	−0.094^⁎⁎⁎^ (s.e. 0.033)	−0.096^⁎⁎⁎^ (s.e. 0.033)	−0.112^⁎⁎⁎^ (s.e. 0.041)
Hospitalization	−0.168^⁎⁎⁎^ (s.e. 0.04)	−0.15^⁎⁎⁎^ (s.e. 0.047)	−0.153^⁎⁎⁎^ (s.e. 0.044)	−0.185^⁎⁎⁎^ (s.e. 0.054)
Deaths (confirmed)	−0.204^⁎⁎⁎^ (s.e. 0.066)	−0.181^⁎⁎^ (s.e. 0.075)	−0.181^⁎⁎^ (s.e. 0.072)	−0.264^⁎⁎⁎^ (s.e. 0.085)
Fixed effects	Yes	Yes	Yes	Yes
Demographic controls	No	Yes	Yes	Yes
Health controls	No	No	Yes	Yes
Comorbidity controls	No	No	No	Yes

Note: *p<0.10, **p<0.05, ***p<.01.

### Sensitivity analyses

We explored the sensitivity of these state-level results in two dimensions. Specifically, we truncated the sample at varying time points, and we also sequentially excluded states according to levels of underreporting. With regard to reporting delays, we found that about half of cases are reported immediately, and 80% are reported within three days. However, over 10% are not reported within a week and 5% are only reported after three months or more. As the final weeks of the individual-level data may be less complete than earlier weeks, we explored different cutoff dates for data inclusion. We found a small decline in the impact of insurance on mortality and cases over time, but a rise in the impact on hospitalization (Table 4).

**Table 4:** Effect of alternative cut-off dates. Proportion change in COVID-19 outcome variables when only data reported by a particular date are included, to examine the potential impact of incomplete reporting on our analysis.

Cutoff date	Number of symptomatic people delaying test	Cases	Hospitalization	Deaths
5/9/2020	-0.019	-0.209***	-0.140***	-0.233**
6/18/2020	-0.291***	-0.181***	-0.148***	-0.239***
7/28/2020	-0.361***	-0.163***	-0.155***	-0.249***
9/6/2020	-0.294***	-0.148***	-0.166***	-0.265***
10/16/2020	-0.209***	-0.135***	-0.176***	-0.272***
11/25/2020	-0.116*	-0.124***	-0.182***	-0.27***
1/4/2021	-0.053	-0.117***	-0.185***	-0.267***
Full sample (through February 13, 2021)	-0.017	-0.112***	-0.185***	-0.264***

Note: *p<0.10, **p<0.05, ***p<.01.

The association between health insurance and testing delays falls over time (Table 4). Over the entire period of our analysis, insurance coverage is associated with a small but not statistically significant increase in the number of symptomatic individuals who have not been administered a test. However, universal insurance coverage is associated with a 35.9% reduction in the days that symptomatic people remain untested when we look only through the summer of 2020 (Table 4).

We considered the impact of sequentially excluding states according to the extent to which COVID-19 data are under-reported to the CDC. We determined the extent of under-reporting based on the percentage fewer deaths in the individual-level data procured from CDC compared with data reported by state health departments. As states with substantial under-reporting are sequentially excluded, we found an increasing impact of health insurance on hospitalization and mortality, and a reduced impact on cases (Table 5).

**Table 5:** Effect of dropping states from analysis because of limited reporting. Proportion change in COVID-19 outcome variables with various sets of states, according to their completeness in reporting.

Number of states included	14	16	21	29	34	50
Screen (states included only if fewer than % deaths missing)	5%	10%	20%	40%	80%	100%
Testing delays	-0.076	-0.047	0.061	-0.007	-0.013	-0.017
Cases	-0.067	-0.059	-0.027	-0.053	-0.044	-0.11***
Hospitalization	-0.423***	-0.449***	-0.383***	-0.32***	-0.26***	-0.19***
Deaths	-0.571***	-0.623***	-0.497***	-0.41***	-0.34***	-0.26***

Note: *p<0.10, **p<0.05, ***p<.01.

### Contiguous counties analysis

**Figure 3:**
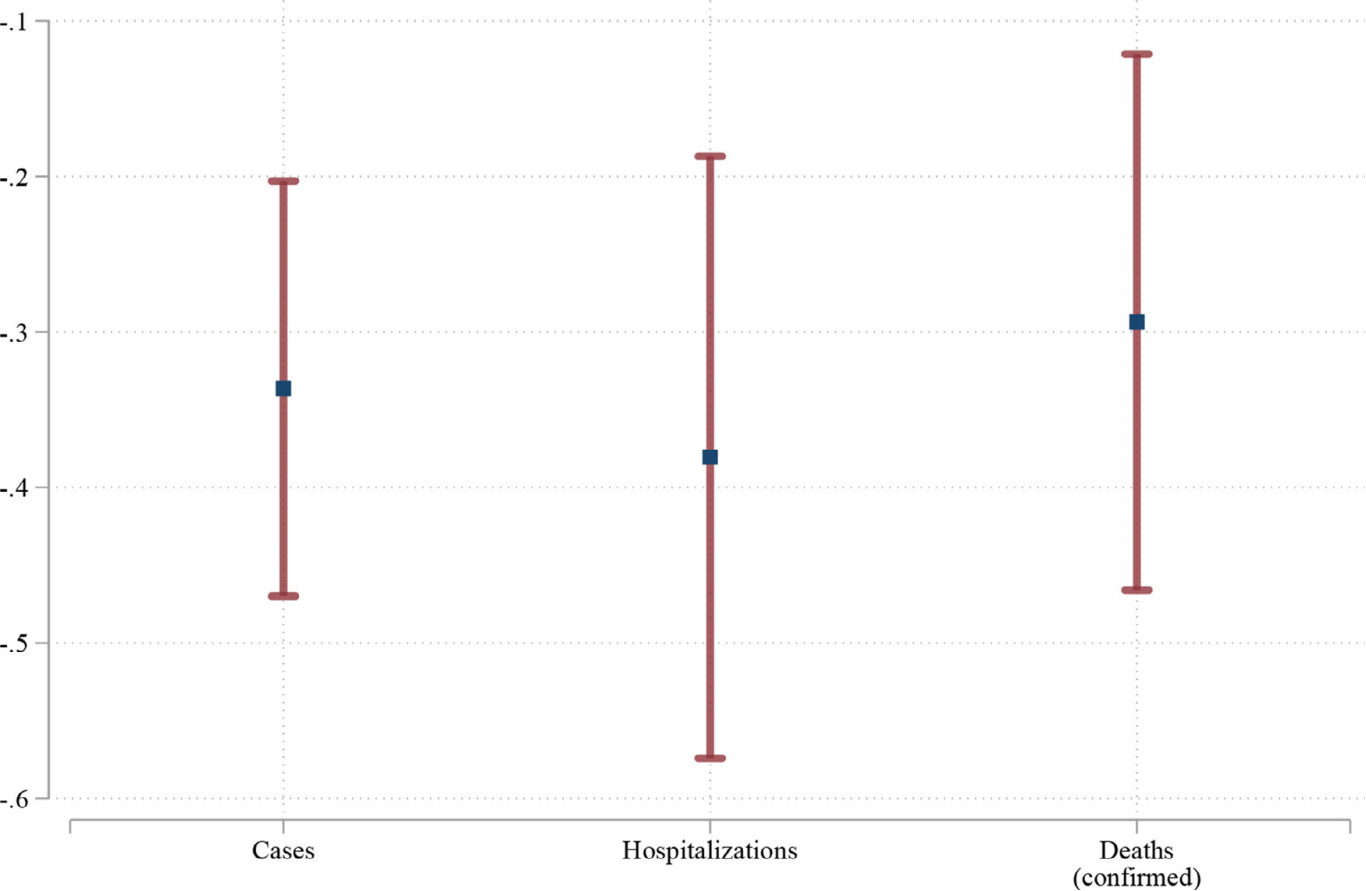
Effect on COVID-19 outcomes of moving from current health insurance rate to full coverage using contiguous counties analysis. The blue dots show the predicted effect as a percentage change in each outcome; the red bars show the 95% confidence intervals.

We evaluated the robustness of our findings by comparing adjacent counties across state lines where one state has expanded Medicaid under the Affordable Care Act and the other has not. This contiguous county analysis supports the state findings that high health insurance coverage is associated with a significant reduction in cases, hospitalization, and mortality (Table 6). Under this design which controls for county characteristics that are similar across state lines, moving to full insurance coverage from the average rate of 89% would be associated with 30.9% reduction in cases, 36.1% reduction in hospitalizations, and 27.7% reduction in mortality (Table 6; Figure 3). These results, furthermore, are all significantly different from zero at the 99% level. Our base case analysis was performed using counts of COVID-19 outcomes; however, the associations are much weaker when outcomes are regressed on a per capita basis. Under that analytical framework, only the cases show a reduction that is significant at 95% confidence.

**Table 6:** Sensitivity analysis comparing contiguous counties with and without Medicaid expansion. This table provides the proportion change in four outcome variables based on two-stage regressions for contiguous counties defined as physically adjacent counties in states with and without Medicaid Expansion. The first-stage regression is for the county rate of health insurance including the state-Medicaid-Expansion; the second stage is for the COVID-19 outcome variable. Two sets of regression results are reported, those where the dependent variable is the number of cases, the other where the dependent variable is the percentage of the population. Data are through mid-February 2021.

	Counts	Per Capita
Cases	-0.309^⁎⁎⁎^ (s.e. 0.075)	-0.059^⁎⁎^ (s.e. 0.022)
Hospitalization	-0.361^⁎⁎⁎^ (s.e. 0.098)	-0.009 (s.e. 0.095)
Deaths (confirmed)	-0.277^⁎⁎⁎^ (s.e. 0.091)	0.05 (s.e. 0.049)

Note: *p<0.10, **p<0.05, ***p<.01.

## Discussion

COVID-19 overwhelmed the US healthcare system during the initial phase of the pandemic and within local intermittent surges. Our analysis suggests that frailty in the financing structure of the system itself weakened pandemic mitigation, increasing the need for critical care and raising mortality. Deploying two independent analytical frameworks, we found that wider insurance gaps exacerbated local COVID-19 outbreaks and resulted in more cases, hospitalization, and death than experienced by jurisdictions with better coverage. Reducing the number of Americans without health insurance is a crucial and underappreciated component of pandemic preparedness.

One study limitation is our use of 2015-2019 health insurance coverage. Our estimates of the COVID-19 mortality attributable to the lack of insurance coverage may therefore be conservative, because the pandemic also fueled a decline in health insurance coverage. In our main analysis, we estimated the national change in COVID-19 outcomes that would be expected if the nation had perfect coverage, compared to that 2015-2019 coverage. However, the economic repercussions of the pandemic led to loss of employment and therefore employer-provided health insurance for millions who had been previously covered. Data from Medicaid-SCHP and for employment-based health insurance enrollment from the Bureau of Labor Statistics indicate that health insurance coverage fell by 10% in April 2020, with a 15% loss of employment-based enrollment balanced by a 2% increase in Medicaid enrollment.^14^ This widened coverage gap implies that a larger proportion of COVID-19 cases, hospitalizations, and deaths may in fact be attributable to a lack of insurance. Once employment and insurance data for the pandemic period become available, an analysis based on changing coverage over time would be a valuable contribution.

A second limitation is that our control for structural effects is at the state level, and therefore our measure of the effect of insurance may also be capturing some of the structural effects of living in localities *within* states with low insurance coverage.^15^ In particular, low-wage and precarious jobs may also have heightened workplace exposure risk.^16^^,^^17^ While our fixed-effect approach controls for these other factors where they are constant, low insurance coverage may also be associated with other conditions correlated with elevated mortality, such as high rates of poverty or lack of social services. Such conditions would likewise be expected to increase with the economic disruption attributable to the pandemic.^18^^,^^19^ For COVID-19, these may be important modifiers, inflating our estimate of the effect of insurance.

Our contiguous county estimates leverage variation in Medicaid eligibility thresholds between adjacent counties saddling state borders, a common approach for evaluating Medicaid expansion.^20^, ^21^, ^22^, ^23^ This research design is not only intuitive, as adjacent counties tend to be similar, but also has a clear identifying assumption. By including pair-specific time effects and county effects, this design controls for all time-invariant characteristics within each county and all time-variant characteristics that are stable within each pair.^24^ Hence, this strategy uncovers the effect of generous Medicaid policies on COVID-19 outcomes if unobserved characteristics are stable within each pair. One disadvantage of this approach is that state-level political differences may be a threat to identification, correlating with both a state's decision to expand Medicaid and their response to the COVID-19 pandemic. We mitigate this limitation by including relevant time-variant controls such as COVID-19 testing.

We included the prevalence of comorbidities as a control in order to rule out the possibility that differences in COVID outcomes are attributable to differences in underlying health status of the population. If high insurance coverage is associated with fewer comorbidities, then controlling for comorbidities should reduce the magnitude of effect that insurance coverage has on COVID-19 outcomes. Instead, we find the opposite. This may be due to the fact that those with insurance are more likely to have a diagnosis of their comorbidity than those who are uninsured. More research is warranted to investigate the individual-level relationship between insurance status, comorbidities, and COVID-19 outcomes.

We found that the effect of insurance coverage on cases, hospitalizations, and deaths was consistent even when we shifted the cutoff date for data inclusion. For testing, the association with healthcare coverage was sensitive to cut-off date and there is evidence that insurance coverage had a greater effect on testing early in the pandemic. Through the summer of 2020, insurance coverage was associated with shorter testing delays as well as fewer symptomatic people waiting for a test. Reduced testing delays curtail disease spread, as both isolation of the infected person and the contact-tracing process are initiated earlier. For the infected person, faster diagnosis can lead to earlier treatment, thereby reducing the risks of hospitalization and mortality. Legislation mandating that COVID-19 testing is to be provided free for all including no liability for uninsured individuals was enacted in the March 2020 Family First Coronavirus Response Act and the April Coronavirus Aid, Relief, and Economic Security Act.^25^ However, anticipated cost sharing and a lack of regular connection with a primary care provider likely still contributed to delays in test-seeking among those without health insurance in the early months of the pandemic. Increased awareness and accessibility of free testing ameliorated the association between insurance coverage and testing delay by the end of 2020.^26^

Federal law subsidizing both testing and medical care for the uninsured with COVID-19 did not erase the association between insurance coverage and COVID-19 disease outcomes. Among the potential reasons for this durable effect are the loopholes that continued to exist, such as charges to patients for aspects of their care that were not specific to COVID-19. Such practices deter patients, as does a lack of communication about the federal subsidy.^27^ Furthermore, uninsured patients with non-specific initial symptoms may delay care, as treatment for an etiology other than COVID-19 would not be covered by the federal law. Concurrently, hospitals and some providers might still prefer privately insured patients over the uninsured covered by federal law because of higher reimbursement rates.^28^ Strikingly, we found that low insurance coverage remains associated with increased hospitalization despite both patient and provider financial disincentives for hospitalization of the uninsured. One contributing factor is that delays in care can exacerbate COVID-19 severity.^29^ Beyond the direct individual-level pathway from hospitalization to death, the effect of additional hospitalizations on an overwhelmed health care system also contributes to higher mortality among both uninsured and insured individuals in the locality.

By demonstrating the adverse impact of incomplete insurance coverage on COVID-19 caseloads and mortality, this analysis highlights the limits of our reliance on this system of market-driven healthcare. Pervasive concerns over cost and lack of connection to healthcare providers are not restricted by any means to COVID-19. These systemic problems plague America's healthcare across all diseases, contributing to tens of thousands of unnecessary deaths each year even before the COVID-19 pandemic.^13^ The implementation of a single-payer, universal healthcare system, such as that proposed by the Medicare for All Acts of 2019 ^30^ and 2021,^31^ would mitigate the burden of both the COVID-19 pandemic specifically and healthcare disparities more broadly in the United States.

## Declaration of interests

None.
